# African swine fever at the wildlife-livestock interface: challenges for management and outbreak response within invasive wild pigs in the United States

**DOI:** 10.3389/fvets.2024.1348123

**Published:** 2024-01-26

**Authors:** Vienna R. Brown, Ryan S. Miller, Kim M. Pepin, Keith M. Carlisle, Merril A. Cook, Cole F. Vanicek, Lindsey K. Holmstrom, Lisa T. Rochette, Timothy J. Smyser

**Affiliations:** ^1^United States Department of Agriculture, Animal and Plant Health Inspection Service, Wildlife Services, National Feral Swine Damage Management Program, Fort Collins, CO, United States; ^2^United States Department of Agriculture, Animal and Plant Health Inspection Service, Veterinary Services, Center for Epidemiology and Animal Health, Fort Collins, CO, United States; ^3^United States Department of Agriculture, Animal and Plant Health Inspection Service, Wildlife Services, National Wildlife Research Center, Fort Collins, CO, United States; ^4^Colorado State University, Human Dimensions of Natural Resources Department, Fort Collins, CO, United States; ^5^United States Department of Agriculture, Animal and Plant Health Inspection Service, Veterinary Services, National Preparedness and Incident Coordination, Riverdale, MD, United States; ^6^United States Department of Agriculture, Animal and Plant Health Inspection Service, Veterinary Services, Aquaculture, Swine, Equine, and Poultry Health Center, Swine Health, Raleigh, NC, United States

**Keywords:** African swine fever, domestic-wildlife interface, emergency response, management implications, policy

## Abstract

African swine fever (ASF) causes significant morbidity and mortality in both domestic and wild suids (*Sus scrofa*), and disease outbreaks convey profound economic costs to impacted industries due to death loss, the cost of culling exposed/infected animals as the primary disease control measure, and trade restrictions. The co-occurrence of domestic and wild suids significantly complicates ASF management given the potential for wild populations to serve as persistent sources for spillover. We describe the unique threat of African swine fever virus (ASFV) introduction to the United States from epidemiological and ecological perspectives with a specific focus on disease management at the wild-domestic swine interface. The introduction of ASF into domestic herds would require a response focused on containment, culling, and contact tracing. However, detection of ASF among invasive wild pigs would require a far more complex and intensive response given the challenges of detection, containment, and ultimately elimination among wild populations. We describe the state of the science available to inform preparations for an ASF response among invasive wild pigs, describe knowledge gaps and the associated studies needed to fill those gaps, and call for an integrated approach for preparedness that incorporates the best available science and acknowledges sociological attributes and the policy context needed for an integrated disease response.

## Introduction

African swine fever (ASF) causes significant morbidity and mortality in swine (*Sus scrofa domesticus*) and can cause profound economic costs for the pork industry due to death loss, the cost of disease control, and trade restrictions imposed on ASF positive regions (1). Additionally, this disease impacts animal welfare, rural development, and food security across local, national, and international markets (2). Managing this hemorrhagic virus in domestic swine exclusively is challenging and complex; however, wild boar (*S. scrofa*) and invasive or feral suids are also susceptible to African swine fever virus (ASFV) and are now recognized to play an important role in the spread and maintenance of ASFV throughout affected regions (3, 4). The potential for ASFV transmission across the wild-domestic interface necessitates a holistic approach for disease management to prevent wild or feral populations from posing a persistent threat of disease spillover, particularly for countries with the risk of large economic consequences if the disease is not controlled (5, 6).

African swine fever virus is a large double stranded DNA virus in the family *Asfarviridae* (7, 8) that exclusively impacts members of Suidae (9). ASFV is transmitted through direct and indirect contact and can be vectored through competent soft-bodied ticks of the *Ornithodoros* genus (10). Numerous strains of ASFV can be found across the globe with clinical presentation ranging from mild to severe, although most of the strains currently circulating in epizootic regions cause moderate to severe disease. Infected swine typically develop a high fever, inappetence, and lethargy with most (~95%) animals succumbing within a week of infection (10). ASFV is endemic across most of the African continent, Eastern Europe, China, and much of southeast Asia (11); however, in recent years other parts of Europe have experienced ASFV outbreaks including Belgium in 2018 (12) (although Belgium has since eradicated the virus) (13); Germany in 2020 (14); Italy in 2022 (15); and Sweden in 2023 (16). Additionally, ASFV was identified in the Western Hemisphere for the first time in nearly 40 years with an ongoing outbreak on the island of Hispaniola (representing the counties Dominican Republic and Haiti) since 2021 (17). Aside from the acute lethality and the numerous source populations for ASFV distributed across the globe, there are several other attributes of ASFV that make it a particularly challenging pathogen to contain and control.

ASFV poses a significant threat to global food security and nutrition as 113 million tons of pork were consumed in 2022 (18). In addition to the production losses and morbidity/mortality caused by the virus, outbreaks of ASFV have significantly altered global export markets for pork products and have negatively impacted the swine industry in affected countries (19). The economic impacts of an ASFV introduction to the U.S. would be significant considering 27.5% of U.S. pork production was exported in 2022, representing a US$7.7 billion economy (20). An ASFV detection in either domestic or wild swine populations could trigger a halt to export activities, and the time needed to recover some or all exports is unknown and would be largely dependent upon the scale of the outbreak. Preliminary estimates suggest losses to the U.S. pork industry could be US$15 billion and US$50 billion for 2- and 10-year scenarios, respectively (21). Given the unique risk wild suids pose as a source for ASFV spillover to domestic herds, we describe the challenges for the control and management of this pathogen among invasive wild pigs from epidemiological and ecological perspectives and identify knowledge gaps that could complicate an effective outbreak response.

### Challenges of disease control among domestic populations

Pathogens at the livestock-wildlife interface are unique in that the spillover-spillback dynamics create their own epidemiological scenario that are often not well understood (22). The response plan to contain and control ASFV among domestic pigs in the U.S. establishes biosecurity procedures that swine producers are expected to follow during an ASFV event to prevent transmission. Additionally, individual states may also impose additional biosecurity requirements. As a primary means for control, the response plan establishes a 5-km control area and a minimum of a 5-km surveillance zone around ASFV affected domestic swine premises (i.e., domestic pig production operations) as well as around infected wild pigs or wild pig carcasses (23, 24). Within this response zone, pathogen control and surveillance activities would be targeted and prioritized. Regardless of whether only domestic swine or only wild pigs are affected, all domestic swine premises within the control area would be subject to quarantine, movement restrictions, permitted movement requirements, and surveillance due to the potential risk of exposure and transmission. Domestic swine premises located in the surveillance zone—the movement-free area (hereafter, free area) surrounding the control area—would not be under quarantine/movement restrictions but would be subject to enhanced surveillance and biosecurity requirements.

Depending on the geographic region of the outbreak, the movement restrictions to domestic swine for premises located in a control area, even if the outbreak is restricted to wild pigs, could have significant implications for animal welfare. Specifically, the commercial swine industry is highly vertically integrated, requiring regular movements among different production stages (25), with most animals moving from farrowing to finishing to slaughter in the same cohort (26). Production facilities are designed for specific stocking densities for animals of a certain body size and interruptions in the supply cannot be readily absorbed (27). Thus, should movement restrictions for domestic swine be imposed due to an ASFV outbreak in wild pigs, producers may be unable to move animals to slaughter, which may necessitate euthanasia and carcass disposal at the production facility or risk animals experiencing welfare concerns due to their growing too large to live comfortably in the available production space. Thus, an outbreak of ASFV that soley occurs in wild pigs can still result in significant economic impacts to the domestic swine industry.

The primary means to control an ASFV outbreak involving domestic or wild swine will be culling of infected, exposed, or at-risk animals (28). Culling can be logistically intensive and costly, depending on the size of an outbreak. In the event that an outbreak involving domestic swine cannot be controlled using culling, vaccination will potentially be an important strategy to control a large outbreak of ASF in countries that wish to maintain export markets.

Development of an efficacious vaccine against ASF has been challenging. The virus is very large (170–190 kb), complex, and encodes many proteins that evade the host immune response, all of which have complicated vaccine development (29). Additionally, the key determinants of host protection have been difficult to elucidate (30). Improvements in vaccine development are encouraging, although hurdles remain for the development of a fully licensed DIVA (differentiation of infected from vaccinated animals) compatible vaccine that is available for broadscale use in the U.S. (31). Recently a live-attenuated, DIVA compatible vaccine (ASFV-G-ΔI177L) has been shown to be safe and highly efficacious (32–34). This candidate vaccine is currently being used in Vietnam and the Philippines to control ASF. While this candidate vaccine shows promise, how it will perform during an outbreak to control ASF transmission in the presence of wild suids serving as a source for repeated spillover remains unknown. Thus, in the absence of a commercially available, effective vaccine approved for emergency use in the U.S., virus eradication is the only current strategy for ASFV management.

Another significant challenge for control of ASFV is virus resilience (35, 36). ASFV has been shown to be uniquely resistant to environmental conditions, remaining viable in pork throughout common curing processes and is stable across a broad range of pH levels and temperatures (37). Additionally, the virus is disseminated throughout the body of the host over the course of infection; thus, all secretions, excretions, and tissues contain virus (38). Swill feeding, the practice of feeding food scraps and other waste to swine, is common among smallholder pig operations worldwide and provides an important pathway for ASFV transmission. In fact, contaminated swill has been implicated as an important route of transmission in numerous ASFV outbreaks, globally (39). Garbage feeding is regulated in the U.S. through the Swine Health Protection Act, requiring producers that engage in the practice to obtain a license and adhere to appropriate cooking and handling of garbage feed for swine [(40); (Public Law 96–468)]. Additionally, the Swine Health Protection Act, allows states within the U.S. to further regulate garbage feeding with 23 states fully prohibiting the practice. The capacity for ASFV to be readily transmitted through pork-based products, especially food waste, and resilience to typical curing processes could contribute to the risk of anthropogenic viral movement. In addition to contaminated products containing infectious virus, carcass-based ASFV transmission amongst wild boar and between wild boar and domestic swine (41) also serves as a route of transmission. Disposing of ASFV-infected carcasses is very challenging (42); however, it appears to be important for controlling an outbreak (43).

### Challenges of disease control among wild populations

Wild pigs (also commonly referred to as feral swine) are an invasive species that are non-native to North America (44). Widespread and abundant populations of invasive wild pigs, particularly through Texas and the southeastern region of the U.S., could increase the complexity of achieving disease control or elimination in the event of an ASF outbreak. However, achieving control and elimination of ASF in wild pigs is a particularly important objective to limit potential economic consequences. The European experience has demonstrated that once ASF is established in free-living suids (i.e., wild boar in this context), control becomes increasingly difficult (41) and even a small outbreak in wild pigs is expected to incur large economic impacts (45).

Effective surveillance is critical for early detection and subsequent control of a foreign animal disease (FAD) introduction. Delays in detection can result in significant increases in outbreak size, severity, duration, and the likelihood that ASFV persists in wild pigs (46). For example, ASFV was likely circulating in wild boar in Asia well before it was detected (47). These factors have prompted proactive ASF surveillance in wild pigs in the U.S. to shorten time-to-detection (48).

## Unique risk posed by ASF establishment in wild pigs

**Figure 1:**
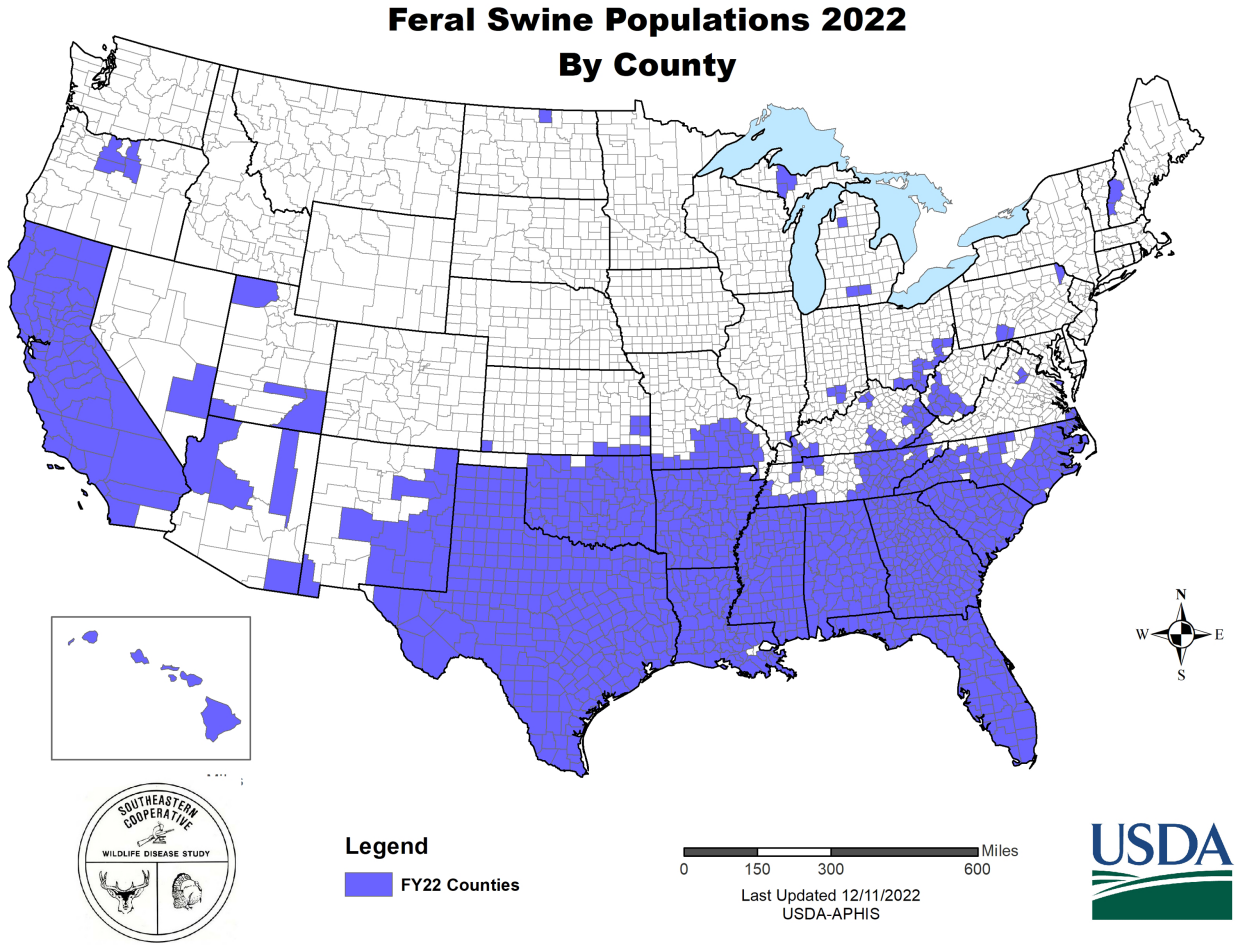
Spatial extent of wild pigs in the United States in 2022.

In the U.S., wild pigs are characterized as any free-living suid regardless of whether the ancestral origins of an individual pig are that of domestic swine or Eurasian wild boar; however, genetic analysis has demonstrated that the vast majority of animals removed from invasive populations are hybrids of domestic and wild lineages (49, 50). The invasive potential of wild pigs is well established as they are a highly adaptable, generalist species with uniquely high reproductive rates given their body size (51)—all attributes that contribute to wild pigs being characterized as among the worst invasive species in the world (52). Wild pigs are broadly distributed with self-sustaining populations established across many U.S. states and territories [Figure 1; (53)]. Further, environmental and climatic models indicate that much of the U.S. is suitable habitat and, thus, susceptible to wild pig invasion (54). The broad distribution of invasive wild pigs would increase the complexity of achieving disease control or elimination in the event of a ASF outbreak—reflective of the European experience in which ASF has become established among native wild boar—with abundant populations of a free ranging suids serving as a source for ASFV and representing a persistent spillover threat. Although ecologically similar, management of native wild boar as compared to invasive wild pigs have some innate differences in that a stated management objective of elimination may be socially acceptable for invasive species (55). Accordingly, to protect domestic herds from ASFV introduction and/or establishment, an integrated plan is needed that considers the importance of managing ASF among wild populations.

Managing wild populations for control of a foreign animal disease (FAD) such as ASF is fundamentally different than other wildlife disease control programs in North America, which have focused on managing risks associated with chronic endemic diseases (e.g., brucellosis, bovine tuberculosis, rabies, or chronic wasting disease). Containing and controlling an ASF outbreak at the landscape scale first requires identifying the presence of ASFV, through active or passive surveillance of wild pigs, as early detection is essential for rapid disease control. However, disease detection can be particularly difficult among wild populations (47). Implementing wildlife surveillance at a national scale is highly complex and the resulting data can pose challenges for inferring epidemiological parameters (e.g., prevalence) (56). To help mitigate these issues, an adaptive risk-based surveillance approach has been adopted for FAD surveillance in wild pigs in the U.S. (48). As a component of ongoing population control efforts conducted throughout the extent of the invaded range, samples for ASFV surveillance are being collected from apparently healthy wild pigs that are lethally removed by USDA APHIS Wildlife Services and from federally inspected slaughterhouses prior to commercial sale of pork from wild pigs into national and international markets (57). This targeted approach is responsive to changes in perceived risk over time, as surveillance effort is reallocated annually to reflect a dynamic risk landscape and prioritize those areas deemed to be at the greatest risk of ASFV introduction.

Many of the globally circulating ASFV strains are highly virulent and result in high lethality rates within a week of infection. Therefore, it is likely that there would be epidemiological impacts to infected wild pigs, such as altering movement patterns and social behaviors (58), during both the latency and infectious periods (59, 60). Detecting sick wildlife or their carcasses is extremely difficult due to stoicism (61) and decomposition rates (62), respectively, thus elevating the importance of proactive surveillance. If ASFV were to be detected among wild pigs, expanded surveillance would be conducted to determine the geographic extent of the outbreak (23). Properly balancing what is appropriate, necessary, and feasible within the context of an initial response requires a robust understanding of ASFV epidemiology, wild pig ecology, and logistical constraints of an operational response.

In response to a potential ASF detection among wild pigs, policy developed as a component of FAD preparedness specifies the delineation of a control area comprised of an infected zone (inner most zone that immediately surrounds infected wild pigs) and a buffer zone (zone that immediately surrounds an infected zone). The surveillance zone (zone outside and along the border of a control area) is part of the free area (i.e., areas not included in any control area) (24). These control areas would be defined based on radii surrounding the area where the positive animal(s) was detected. The control area would be adaptive, such that it would be expanded by the same distance to encompass additional detections of infected animals. As general guidance, the established response plan recommends a minimum 3 km radius for the infected zone, 2 km for the buffer zone, and 5 km for the surveillance zone, for a total radius of 10 km from the detection location. Radii defining the control area were determined based on observations of wild pig movement distances (63), wild pig contact distances (64, 65), and domestic swine disease response policies. However, as evidenced from animal movement studies conducted across a breadth of invaded ecosystems (63–65), it is likely that the most effective radii differ based on local population attributes and ecological factors (66).

Upon pathogen introduction, the spatial spread of infectious disease in any host population is driven by contact rates among hosts and the pathway of pathogen exposure. To predict ASF dynamics among wild pigs, it is necessary to consider the social structure of the species and movement patterns as the underlying mechanism that would dictate rates of disease spread (67, 68). Wild swine are highly social with populations organized into matrilineal family groups called sounders (69, 70). Sounders generally consist of one to several adult females and their offspring, with adult males moving among sounders (71). Understanding of the hierarchical structure of local populations and the concomitant contact rates within versus between social groups is, thus, crucial for accurately predicting spatial transmission dynamics of ASFV. Contact rates between sounders is influenced by the home range characteristics and movement patterns of wild pigs. Wild pig movement patterns exhibit two distinct movement processes: (1) short-term, day-to-day movements characterized by a local home range centroid and (2) infrequent long-distance directional movements, well beyond established home ranges, that can occur when resource conditions change or social structure is disrupted, particularly when populations are at low densities (66, 72). Home range attributes and daily movement rates are influenced by population densities and resource availability, which complicates scaling predicted rates of disease spread across the diversity of ecosystems invaded by wild pigs in the U.S. For example, wild pigs require water for thermoregulation, and previous work has shown that wild pigs establish larger home ranges in more arid environments. Thus, a model for predicting local movement behavior and home range centroid shifts over fine temporal scales (i.e., weekly) from factors such as habitat, ecoregion, time of the year, and local density is needed to predict spatial spread of ASFV over a time scale that is relevant to response efforts.

In addition to the natural movements of wild pigs that drive epidemiological dynamics within and between social groups in hierarchically structured populations, genetic analyses have repeatedly demonstrated high frequency of human-mediated translocation for this species, with the potential for translocation to amplify rates of disease spread (50, 73, 74). For example, Tabak et al. (74) and Hernandez et al. (73) leveraged population genetic analyses to delineate genetically cohesive populations and map the movement of wild pigs among those populations in California and Florida, respectively. Tabak et al. (74) identified informative sociological factors associated with both domestic pig production and recreational hunting that were informative in predicting rates of wild pig translocation into and out of California counties. Hernandez et al. (73) determined that holding facilities—intermediate facilities in which live-trapped wild pigs are temporarily held before animals are moved to slaughter—serve as foci in local patterns of translocation, presumably with animals either escaping or being released from these facilities. Smyser et al. (50), working across the invaded range within the contiguous U.S., identified numerous anecdotes in which emergent populations were attributable to long-distance translocations from established invasive population as opposed to the escape or release of domestic pigs. The concern with high rates of human-mediated translocation, regardless of whether the movement is within state boundaries (73, 74) or over much greater distances (50), is that this process could greatly accelerate the rate of disease spread beyond what could be expected from epidemiological processes informed by natural movement patterns and contact rates alone.

As a tool to integrate ecosystem-and population-specific knowledge of movement patterns and contact rates into an ASF response, a spatial disease transmission model was developed based on the epidemiological characteristics of genotype II virus circulating in Europe (66). This epidemiological model was used to evaluate the potential impacts of control area size under different ecological conditions and management intensities. The radial distance delineating the control area was optimized to minimize outbreak duration and distance of spatial spread given reasonable management constraints (e.g., control intensity, local movement and contact ecology, and time of the introduction relative to initial detection). Several different optimal radii were identified depending on local wild pig movement patterns and contact rates, suggesting that understanding how these parameters vary among invaded ecosystems is needed to define the appropriate size of the control area given landscape-and population-specific attributes. Under most conditions, radii of >14 km were needed to rapidly contain an outbreak when initial detection occurred 4 months after introduction, but smaller radii were effective under early detection (<8 weeks after introduction) when high culling intensities (>15% weekly) could be implemented. Disease elimination was generally possible within 22 weeks across the conditions examined, but high control intensities (>10% weekly) were needed to achieve elimination within a year when wild pig movement and contact rates were high.

Modeling efforts highlighted uncertainties in parameters that could improve confidence in predictions of the epidemic duration and spatial spread under different response strategies (66). In particular, feasible rates of removal can vary dramatically depending on local conditions such as ecosystem attributes (e.g., vegetation density or terrain ruggedness), road access, and landownership with potential restrictions for accessing private property. These factors would affect both removal rates and carcass recovery rates. Little information exists to understand realistic removal rates for intense, continuous control within a large control area across different habitats. Relatedly, removal rates may decline as density is reduced as animals may become more difficult to locate at low densities or could increase their daily movement rates (i.e., home range size). Field studies to understand the relationship between density and removal rates could help to reduce uncertainty in elimination time. Also, as it is likely that elimination of ASF would occur before complete elimination of wild pigs in the control area as wild pig abundance falls below a level that can sustain ongoing transmission. Understanding which field-based measures provide the earliest evidence of ASF elimination is needed for efficient determination that an outbreak among wild pigs has been controlled.

In addition to understanding the epidemiological and ecological underpinnings of ASF, human activities are recognized as playing an important role in disease dynamics (75). As such, public outreach and stakeholder engagement are fundamental to any successful management response (76, 77). Drawing from previous experiences of disease outbreaks in wildlife such as with highly pathogenic avian influenza and chronic wasting disease, it is imperative to identify stakeholders and communicate risk prior to an outbreak event (78, 79). Garnering awareness and sociopolitical support in advance of a crisis, allows for a smoother and more rapid transition from preparedness, prior to detection, to a management response following detection. Clear, sustained communication is paramount through all stages of an outbreak, whether eradication is achievable or the response objective is minimizing economic or ecological costs as the disease transitions to endemic status (76).

## Knowledge gaps and management needs

The task for those working on ASF preparedness is to integrate the best available knowledge in the formulation of a response plan that will ensure disease containment and, ultimately, elimination. However, much of the available knowledge pertaining to wild pigs has been collected from routine population control and damage management efforts, which are distinct from intense, continuous removal efforts that would be mobilized in the event of a disease outbreak response. Because mobilizing a simulated FAD response is logistically challenging and very expensive, uncertainty persists in logistical, ecological, and epidemiological aspects of an ASF response.

Various logistical challenges could delay or prolong the elimination of ASF once established among wild pig populations. Landscapes invaded by wild pigs vary in the extent and accessibility of road networks. Road infrastructure differentially influences the feasibility of various control techniques. For example, whole sounder removal efforts implemented with the deployment of large traps is more dependent on road networks in that it is difficult to haul large traps into remote habitats, whereas aerial gunning is far less dependent on road infrastructure. Landownership could represent another logistical constraint in that private properties with potentially infected wild pigs may not be accessible for control activities due to limitations regarding owner permission. Modeling efforts, as described above, could help quantify the epidemiological consequences of heterogeneous land access, at least during the initial stages of a response while permission to access private land would be sought as a component of the integrated and unified response effort. Thus, the operational response to ASF detection will need to be adaptive, tailored for the landscape in which the introduction occurs based on logistical constraints and resources available for control.

An ASF outbreak, with expected high mortality rates and an ensuing management response, in which wild pigs would be removed from the infected zone through intensive culling efforts, would represent an ecological perturbation with uncertainty in the behavioral response of wild pigs. One knowledge gap in the described response plan is how wild pig movement patterns may change in response to rapidly decreasing abundance within the infected zone and/or increased human activity and culling pressure. Boundaries of the delineated control areas and surveillance zones [infected (0–3 km) and buffer (3–5 km) representing the control area, and 5 km for the surveillance zone] are only conceptual for free-living wild pigs unless physical structures are built for containment. Thus, research is needed to quantify the behavioral response of wild pigs to the combination of intense control and potential disease die-offs to elucidate the frequency and distance of animal movements within the control areas. For example, disruptions to social groups due to disease-loss or control efforts could stimulate long-distance dispersal, thus breaching the infected zone (72, 80). Similarly, animals could disperse from the infected zone, fleeing the increased human activity associated with carcass recovery and pressure exerted during control efforts (81, 82). Conversely, wild pigs from surrounding habitats may enter the control area as a result of lower population densities and potentially increased availability of resources, which could increase the burden of culling efforts or rates of disease transmission with increased contact. Integrating understanding of the movement response into a disease spread modeling is needed to inform whether fencing or other similar barriers are crucial for disease elimination in wild pigs.

Identifying and removing carcasses of wild pigs that have succumbed to ASF is another important component of disease control (83) and distinct from routine population control activities that have been used to inform ASF response scenarios. Carcass ground searches—response personnel walking transects through the control area—is labor-intensive and diverts mobilized personnel from other potential response activities. Accordingly, additional tools are needed (e.g., drones, carcass detection dogs) that can be used to efficiently locate carcasses over potentially large control areas. Further, the efficacy (i.e., detection rates) and resources required to implement carcass discovery, regardless of whether those efforts are represented by ground searches or the use of alternative tools, would be expected to vary among ecosystems (in response to vegetation characteristics and topography) and with wild pig densities. Thus, field studies are needed to quantify resources needed and detection rates of carcasses distributed across diverse landscapes in a manner that simulates an ASF outbreak. However, the frequency in which wild pigs contact carcasses (thus posing a transmission risk) throughout the decay process [e.g., (84)] and understanding how contact rates vary across environmental conditions and wildlife communities are elusive. Field studies to resolve these processes help identify effective response strategies for a disease system in which carcasses contribute to transmission. Results of these field studies can then be used to improve disease spread modeling scenarios and evaluate whether the resources invested in carcass removal positively contribute to disease containment and elimination or whether those response resources would be better allocated to other activities (e.g., population control or fencing).

In addition to those wild pigs that may be succumbing to ASF, population control efforts represent a second source of carcasses that will require management, as some animals removed through culling efforts may be infected with ASF. Established methods for carcass disposal in response to mass culling are largely based on production animal settings where animals are concentrated at a single location (e.g., from a single production barn). In the context of an ASFV response among wild pigs, the animals culled as a part of control efforts will be distributed throughout the control area (e.g., 5 km radius surrounding all positive detections). Further, some removal techniques, such as aerial gunning, do not involve direct contact with the animals and would require additional effort for carcass recovery. Thus, additional consideration will need to be given to carcass management of those animals removed from the control area through culling.

As an ASF response progresses, the stated goal is to contain and ultimately eliminate the disease from the affected population, yet substantiating disease freedom poses a distinct challenge. Further, substantiating the absence of disease after an outbreak has been controlled is vitally important for reestablishing export markets and resolving impacts to markets affected by an outbreak. Typical approaches for substantiating disease freedom rely on sampling sufficient numbers of animals to provide high levels of confidence (e.g., 95% certainty) that the disease, if present, is below a given prevalence (e.g., 1% infection rate). This is complicated by spatial dynamics of wild pig populations that are likely to have heterogeneous densities across space and may demonstrate increased and perhaps unpredictable movement patterns after large reductions in abundance. These challenges for substantiating disease freedom in wild populations, using approaches typically applied in domestic animals, will require the development of novel statistical methods that can integrate multiple lines of evidence to determine when an ASF outbreak has been controlled.

In the U.S., regulation of wild pig-related activities primarily falls under the jurisdiction of the states rather than the federal government. State legislatures and agencies have taken a variety of policy and management approaches to wild pig populations that range from population elimination to mitigating damage while maintaining recreational hunting opportunity (85, 86), and this has resulted in a diversity of state regulatory approaches (87). States differ, for example, in the extent to which they allow activities such as wild pig hunting, possession, transport, and release of the animals (88). Additionally, the types of regulatory authorities with responsibility for wild pigs also vary by state and may depend in part on how the animals are classified (i.e., “game” or “nuisance species”) (86) and in some states, there may even be multiple agencies with limited scopes of authority over wild pigs. This variability among states has resulted in a complex and sometimes difficult-to-decipher regulatory landscape that will impact what agency takes the lead on controlling an ASF outbreak as well as what is permitted when conducting control operations. Thus, defining the regulatory environment on a state-by-state basis is an important, but easily overlooked aspect, of preparedness as a response that spans state borders is plausible while coordination and efficient communication will be essential for the success of the response effort.

## Policy as a tool for management/disease protection

Given that the ASFV can be readily transmitted from direct and indirect contact and available vaccines remain in early stages of development, quarantine and movement restrictions for exposed and infected domestic swine and their products is incumbent for successful ASF management (89, 90). The global ASF epidemic has demonstrated that the involvement of wild suids greatly increases the epidemiological complexity of the outbreak (91). The presence of wild pigs in the U.S. adds an additional layer of regulatory complexity largely due to jurisdictional responsibility that is distributed among various local and federal agencies. The rules governing what can and cannot be done with wild pigs varies on a state-to-state basis as does the entity with jurisdiction over regulatory enforcement. For example, approximately half of U.S. states have “no tolerance” policies when it comes to the transport of wild pigs, prohibiting any and all manner of transport, while most of the remaining states allow transport to approved locations and/or under specified conditions (86). As this relates to ASF-related risk, one may reasonably infer that states with more permissive wild pig transport laws and related policies (e.g., allowance of wild pig hunting preserves and slaughter facilities) and larger wild pig populations would have a relatively greater risk of ASF spatial spread through human-mediated movement of the animals. Among such states, Texas stands out for both the size of its wild pig population and the extent of its wild pig transportation and use networks, as described below.

### Nested case study: Texas wild pig movement

Texas has the largest number of wild pigs of any U.S. state, with an estimated population of at least 2.5 million (51). The state also has a deeply entrenched wild pig hunting culture and mature industries (e.g., meat processing and related transportation infrastructure or services associated with recreational hunting or live-capture for slaughter) that profit off the species’ abundance (92, 93). Although Texas funds wild pig population control efforts to mitigate damages suffered by agricultural producers and landowners (94), state policies also accommodate certain wild pig-related interests. For example, Texas allows recreational hunting of wild pigs year-round, including at fenced hunting preserves, and it permits limited and regulated holding and transport of live wild pigs (4 Tex. Admin. Code § 55.9). This is in addition to the unknown but possibly large volume of illegal transport of wild pigs by individuals who wish to release them into uninvaded areas or to augment existing populations for recreational hunting (95).

If ASF were to emerge in Texas, the state-sanctioned pathways for holding and transporting wild pigs (referred to herein as “wild pig market chains”) would present a risk of ASF spread on account of, among other things, the possibility of escapes and improper carcass disposal. To gain a better understanding of wild pig market chains in Texas, including their regulation, eleven individuals from relevant federal and Texas agencies were interviewed, including the Texas Animal Health Commission (TAHC), the USDA-Food Safety and Inspection Service (FSIS), USDA-APHIS-Veterinary Services (VS), and the Texas Department of State Health Services (DSHS). Additionally, federal and state statutes and regulations that bear upon wild pigs in Texas were analyzed, and relevant news reports and published literature was reviewed.

In Texas, the TAHC is primarily responsible for regulating wild pig market chains. Its regulations permit individuals who capture wild pigs to transport them directly to approved holding facilities, authorized hunting preserves, and recognized slaughter facilities—i.e., facilities that operate under federal or state meat inspection laws and regulations (4 Tex. Admin. Code § 55.9(b)). Holding facilities are numerous and widespread in Texas—as of July 11, 2023, there were 62 publicly listed holding facilities in 55 cities—and they serve as linkages in wild pig market chains. These holding facilities purchase live wild pigs from individuals, and the facilities are permitted to hold animals for up to 7 days before transporting them directly to another holding station, to a recognized slaughter facility, or licensed hunting preserve (also referred to as captive hunt facilities or shooting preserves). Importantly, the TAHC requires wild pig holding facilities and hunting preserves to maintain records of wild pig transactions and to meet specified biosecurity requirements. For example, they must maintain a swine-proof fence, and holding facilities cannot be located within 200 yards of domestic swine (4 Tex. Admin. Code § 55.9(c) and (d)). Holding station operators are also required to remove and properly dispose of carcasses of wild pigs that die of certain communicable diseases (4 Tex. Admin. Code §§ 55.9(c) and 59.12). While these regulations do not require disposal if the animals are not suspected to have died from a communicable disease, all holding facility operators sign an agreement with the TAHC that requires prompt removal and burial of all feral swine carcasses.

With regard to wild pig slaughter facilities, there are three general categories in Texas: (i) custom exempt slaughter facilities that process swine for the use of the owner; (ii) state-inspected slaughter facilities, which process swine intended for sale within Texas; and (iii) federally inspected slaughter facilities, which slaughter and process swine intended for domestic and overseas markets. However, only federally inspected facilities typically accept and slaughter live wild pigs in Texas. Akkina et al. (57) reported that between January 1, 2017 and January 4, 2020, the six federally inspected facilities in Texas slaughtered 239,338 wild pigs, which represented nearly 99% of all wild pigs slaughtered in the U.S. at federally inspected facilities during that period (57). According to interviewees with direct knowledge, two of the six Texas facilities slaughter and process the vast majority of wild pigs. Given the immense size of Texas and the relatively small number of federally inspected slaughter facilities, wild pigs may be transported over long distances, including entering Texas across state borders. An interviewee familiar with one facility indicated that it regularly receives wild pigs transported from Oklahoma, more than 110 km to the north. With the stress of trapping and transport, it is not uncommon for wild pigs to become sick or expire before they reach slaughter. Another interviewee reported that at one facility, wild pigs often arrive stressed and in poor health, which is reflected in the relatively high rate of condemnation reported by Akkina et al. (57) between 2017 and 2020.

All wild pigs at federally inspected facilities receive an antemortem inspection and a follow-up inspection by a veterinarian for animals labeled “suspect” (57). It is imperative that wild pigs at slaughter facilities are monitored for signs of foreign animal diseases and are part of a comprehensive surveillance program. Wild and domestic swine at slaughter facilities are targeted for surveillance in the U.S. as part of the integrated surveillance plan for swine hemorrhagic fevers (i.e., African and classical swine fever) (96).

As the foregoing suggests, there is a well-developed regulatory and organizational infrastructure in Texas to support a large network of wild pig market chains. Although biosecurity requirements imposed by federal and state regulations mitigate the risk of escapes and other paths of disease transmission, they do not completely eliminate the risk. In 2011, for example, a Dallas-Fort Worth news organization reported that approximately 30 wild pigs escaped from a Fort Worth slaughter facility (97). Moreover, the stress and hardship wild pigs experience prior to reaching their final destination increase the likelihood of mortalities and improper carcass disposal at holding facilities or during transit. These avenues could have severe consequences, including the loss of domestic swine production, if ASF were to emerge in the wild pig population (98) and would make for an extremely challenging on-the-ground disease management scenario.

## Discussion

The risk of spillover-spillback of ASF at the wild-domestic interface poses a unique challenge for protecting the U.S. domestic pig herd and limiting economic consequences. These challenges are multifaceted, complicated by the biology of the virus, the widespread distribution of wild pigs that could serve as a source of ongoing disease transmission, and diversity of pork production practices. As the global ASF epizootic continues, viral circulation among domestic and wild populations across Africa, Asia, Europe, and on the island of Hispaniola poses risks for introduction into the U.S. because of an increasingly globalized economy and the uniquely resilient nature of the virus. In the absence of effective treatment, the introduction of ASF into domestic herds would elicit a strategic response structured around disease containment, necessary culling, surveillance, and contact tracing. However, effective containment of ASF among invasive wild pigs would require a far more complex and intensive response given the challenges of disease containment among wild populations (99).

In response to threats posed to the pork industry by ASF, a great deal of resources have been invested in developing response plans for a potential ASF introduction, both in the U.S. and many countries across the globe (24). Plans to respond to ASF outbreaks involving wild pigs have been informed with the best available information drawn from ongoing control efforts to reduce population abundance and damage caused by this invasive species (66). However, the potential scale of an ASF response involving wild pigs could be much larger than current control efforts for population and damage reduction. The distinction between past population control activities and planned response efforts highlights knowledge gaps in our understanding of the biological response within the host-pathogen system. How might wild pig movement patterns and concomitant disease transmission dynamics change in response to intensive culling efforts, decreasing densities attributable to both culling pressures and disease-related mortality, and increased human activity associated with carcasses searches/disposal? How can wild pig population densities be efficiently predicted during control efforts to support effective surveillance design and the declaration of post-outbreak disease freedom? Filling these knowledge gaps will require studies that implement consistent, intense control at the scale of a disease response. Modeling exercises have very effectively integrated the available data while delineating the limits of understanding and identifying where assumptions regarding disease dynamics need to be made to continue response planning (66). However, these analyses have also demonstrated that the response of wild pigs, a uniquely generalist and highly adaptable species, varies with landscape context, thus limiting the capacity to generalize across the breadth of invaded habitats. Similar challenges have been reflected in the European experience of managing ASF among wild boar in that management strategies may not be universally effective due to both biological and sociological differences among countries.

In both developing and conducting an effective ASF disease response, it is imperative to not overlook sociological aspects that may impede control. Public education related to biosecurity is an important tool to reduce the risk of initial introduction. During planning phases and throughout an outbreak, public outreach is a critical component of a successful response as a diverse set of stakeholders will be impacted. Education and outreach are essential for generating support among the general public however policy makers have a critical role in establishing a regulatory landscape conducive for an effective response. The spurious description of wild pigs as simply feral domestic animals further confuses jurisdiction of this invasive species (50, 100). The current state-by-state patchwork of policies that regulate wild pigs will need to be integrated into a unified State-Federal Incident Command in the event that a multi-state ASF response is required. Multiple studies have demonstrated ongoing and frequent human-facilitated movement of wild pigs—even into those states that prohibit the possession, transport, or release of wild pigs (50, 73, 74). These translocations have also been linked to the introduction of endemic diseases (i.e., swine brucellosis and pseudorabies) and similarly could function to amplify the spread of ASF. Given the heightened risk of ASF introduction, there is need for improved regulation of movement of wild pigs. As with the extensive research and operational investment into preparation and planning for an ASF response, developing and implementing education, outreach, and policy solutions also represents a lengthy investment. Accordingly, equal urgency and determination is needed in preparing effective strategies for managing the sociological aspects of an ASF outbreak as has been given to biological and logistical concerns.

## Author contributions

VB: Conceptualization, Data curation, Formal analysis, Methodology, Project administration, Supervision, Visualization, Writing – original draft, Writing – review & editing. RM: Conceptualization, Data curation, Formal analysis, Investigation, Methodology, Project administration, Visualization, Writing – original draft, Writing – review & editing. KP: Formal analysis, Investigation, Methodology, Project administration, Writing – original draft. KC: Conceptualization, Formal analysis, Investigation, Methodology, Project administration, Supervision, Writing – original draft. MC: Formal analysis, Investigation, Methodology, Writing – original draft. CV: Conceptualization, Formal analysis, Project administration, Writing – original draft. LH: Conceptualization, Formal analysis, Project administration, Writing – original draft. LR: Conceptualization, Formal analysis, Project administration, Writing – original draft. TS: Conceptualization, Formal analysis, Investigation, Methodology, Project administration, Visualization, Writing – original draft, Writing – review & editing.
